# A Large-Area Nanoplasmonic Sensor Fabricated by Rapid Thermal Annealing Treatment for Label-Free and Multi-Point Immunoglobulin Sensing

**DOI:** 10.3390/nano7050100

**Published:** 2017-05-02

**Authors:** Hana Tzu-Han Lin, Chuan-Kai Yang, Chi-Chen Lin, Albert Meng-Hsin Wu, Lon A. Wang, Nien-Tsu Huang

**Affiliations:** 1 Graduate Institute of Biomedical Electronic and Bioinformatics, National Taiwan University, Taipei 10617, Taiwan; r05945044@ntu.edu.tw (H.T.-H.L.); r02945015@ntu.edu.tw (C.-K.Y.); r05945002@ntu.edu.tw (C.-C.L.); 2 Department of Electrical Engineering, National Taiwan University, Taipei 10617, Taiwan; b04901017@ntu.edu.tw (A.M.-H.W.); lon@ntu.edu.tw (L.A.W.); 3 Graduate Institute of Photonics and Optoelectronics, National Taiwan University, Taipei 10617, Taiwan

**Keywords:** nanoplasmonic biosensing, localized surface plasmon resonance (LSPR), rapid thermal annealing (RTA) Treatment, label-free immunoassay

## Abstract

Immunoglobulins are important biomarkers to evaluate the immune status or development of infectious diseases. To provide timely clinical treatments, it is important to continuously monitor the level of multiple immunoglobulins. Localized surface plasmon resonance (LSPR)-based nanoplasmonic sensors have been demonstrated for multiplex immunoglobulins detection. However, the sensor fabrication process is usually slow and complicated, so it is not accessible for large-area and batch fabrication. Herein, we report a large-area (2 cm × 2 cm) nanofabrication method using physical vapor deposition followed by a rapid thermal annealing treatment. To optimize the sensor performance, we systematically characterized three fabrication conditions, including (1) the deposition thickness; (2) the maximum annealing temperature, and (3) the annealing time. The corresponding absorbance spectrum profile and surface morphology of the nanostructures were observed by a UV-VIS spectrometer and atomic force microscopy. We then tested the sensitivity of the sensor using a glucose solution at different concentrations. The results showed that the sensor with 10 nm gold deposition thickness under 5-min 900 °C rapid thermal annealing can achieve the highest sensitivity (189 nm RIU^−1^). Finally, we integrated this nanoplasmonic sensor with a microchannel and a motorized stage to perform a 10-spot immunoglobulin detection in 50 min. Based on its real-time, dynamic and multi-point analyte detection capability, the nanoplasmonic sensor has the potential to be applied in high-throughput or multiplex immunoassay analysis, which would be beneficial for disease diagnosis or biomedical research in a simple and cost-effective platform.

## 1. Introduction

Localized surface plasmon resonance (LSPR) is an optical phenomenon that occurs when a particular light wavelength interacts with metal nanostructures or nanoparticles and causes resonant oscillation of collective valence electrons nearby. The resonance frequency is strongly related to the refractive index of the environment surrounding the nanostructures, which is affected by surface properties such as the types or amounts of bound molecules. Due to its real-time and label-free nature, LSPR is considered an ideal sensing technique in disease diagnosis [1], drug screening [2], agriculture [3] and environmental monitoring [4,5]. Another important application of LSPR sensing is in immune status monitoring. Cytokines and immunoglobulins are well-known biomarkers used to monitor the symptoms of diseases such as acute and chronic infection, tuberculosis [6], dengue [7], celiac disease [8] or hepatitis A [9]. However, both cytokine and immunoglobulin levels may change quickly as diseases develop. Therefore, previous research endeavors have demonstrated various rapid, dynamic and real-time LSPR sensor-integrated microfluidic devices that can be used for multiplex cytokine [10,11] or immunoglobulin detection [12,13,14]. Such devices can facilitate timely clinical treatments for controlling immune status. Moreover, the simpler equipment footprint, lower sample volumes, and shorter reaction times have made LSPR sensing a promising approach for point-of-care testing.

Recent advancements in nanofabrication and nanomaterial synthesis have made LSPR-based sensor fabrication more accessible. One approach is to use electron beam lithography (EBL) to draw fine metallic nanostructure geometries or patterns [15,16]. The problem with EBL is the low fabrication rate, which is not suitable for large-area (millimeter-scale) nanostructure fabrication. Instead, nanosphere lithography (NSL) [17] and nanoimprinted lithography (NIL) [18] are two alternative fabrication methods for large-area nanostructure fabrication. However, batch nanostructure fabrication is challenging, due to the complicated synthesis processes in NSL and imprinting conditions in NIL. Compared to the above techniques, thermal annealing treatment is a relatively simple and cost-effective method to make large-area nanostructures from a flat metal substrate without a micro- or nanolithography-prepared template. Thermal annealing treatment was originally utilized for releasing the residual stress in metal or semiconductor substrates. Recently, researchers have found that thermal annealing-induced material recrystallization or aggregation could also be used for nanostructure formation [19,20]. One approach is using a pulsed laser to melt thin metal-on-oxide films and create nanoscale islands [21,22]. Another approach is to place a metallic substrate in a furnace for thermal annealing treatment to generate nanostructures. A problem with using thermal annealing to fabricate nanostructures is the randomized size and distribution of the nanoparticles, which may reduce the sensitivity of the LSPR effect due to a flattened absorbance spectrum. Therefore, optimizing the thermal annealing conditions is the key to improving LSPR sensing performance.

In this paper, we demonstrate a simple, rapid, large-area (2 cm × 2 cm) nanostructure fabrication method using physical vapor deposition (PVD) followed by a rapid thermal annealing (RTA) treatment. Compared to thermal annealing, RTA enables extremely high speed heating, allowing the substrate to reach high temperatures (~1000 °C) in a several seconds. Therefore, the completed RTA process only takes 5 min, which is 48 to 96 times faster than previously reported thermal annealing methods (550 °C for 4 to 8 h) [19,23,24]. To optimize the geometry of the nanostructures for the highest LSPR sensing performance, we adjust three fabrication conditions: (1) the deposition thickness, (2) the maximum RTA temperature and (3) the RTA time. We then observe the corresponding absorbance spectrum profiles and surface morphology of the nanostructures using a UV-Visible spectrometer and atomic force microscopy (AFM). To evaluate sensing performance, we test the sensitivity of our nanoplasmonic sensor with glucose solutions at different concentrations. Finally, we integrate the sensor with a microchannel and place it under a spectroscope coupled with a motorized stage for multi-point immunoglobulin (IgG) sensing. By confining the antibody-antigen reaction in a larger surface-area-to-volume ratio microenvironment, the required assay volume is only 60 μL and the antibody-antigen reaction can be done in 50 min. Overall, the novel fabrication method reported in this work constitutes a new way to fabricate a simple and cost-efficient nanoplasmonic sensor with a large sensing area, which can potentially be used for label-free, multiplexed immunoassay studies.

## 2. Experimental Methods

### 2.1. Au Nanostructure Fabrication

To fabricate Au nanostructures, we first deposited thin gold films of various thickness (from 2 nm to 15 nm) on a 0.5 mm microscopic glass substrate (Asahi E-glass e.g., 2000, Ruilong, Miaoli County, Taiwan) by electron beam evaporation (E-beam evaporator, Kao Duen technology, New Taipei, Taiwan) and sputtering (Sputter machine, Kao Duen technology). Both PVD methods provide similar thin film deposition quality for RTA. The deposition rate of E-beam evaporation and sputtering was 0.01 nm s^−1^ at 1 × 10^−5^ torr gas pressure and 0.04 nm s^−1^ at 2.8 × 10^−3^ torr gas pressure, respectively. After thin film deposition, the substrate was placed in a furnace (Mila 5000, ULVAC, Methuen, MA, USA) for RTA. The heating rate was 200 °C s^−1^ and the substrate was cooled to room temperature using a water cooling system.

### 2.2. Spectroscopy and Surface Morphology Analysis

To measure the absorbance spectrum of RTA-treated Au nanostructures, we placed the 2 cm × 2 cm substrate into a customized spectroscopy setup (Figure S1). The system consisted of (1) a broad-spectrum light source, connected to an illumination fiber (UV-VIS 600, Ocean Optics, Dunedin, New Zealand) to excite the Au nanostructures; (2) a spectrometer (HR-4000, Ocean Optics) connected to a detection fiber (UV-VIS 600, Ocean Optics) under the substrate to collect the transmitted light; (3) an *X*-*Y* axis motorized stage to move the substrate and (4) a peristaltic pump (BT100-1F, LongerPump, Hebei, China) to load samples into the microchannel (μ-Slide VI^0.4^, Ibidi) attached to the Au nanostructures.

All spectral data was averaged across 20 spectrum scans with 10 milliseconds integration time using spectrum process software (OceanView, Ocean Optics). The peak intensity and wavelength of the absorbance spectrum were further analyzed by Origin 2015 software. Here, we used an 8-degree (*x*^8^) polynomial curve with *R*^2^ > 0.98 to fit the absorbance spectrum (Figure S2). This fitting function allowed us to determine the peak wavelength and the quadratic coefficient, which corresponds to the full width at half maximum (FWHM). To quantify the surface morphology of the nanostructures, we used AFM (Multi-Mode 8, Bruker, Camarillo, CA, USA) to scan the substrate.

### 2.3. Reagent and Sample Preparation

Glucose (G8270, Sigma-Aldrich, St. Louis, MO, USA) was mixed with deionized water to generate solutions at various concentrations. 98% 11-Mercaptoundecanoic acid (11-MUA; 674427, Sigma-Aldrich, St. Louis, MO, USA), *N*-(3-Dimethylaminopropyl)-*N*′-ethylcarbodiimide hydrochloride (EDC; E1769, Sigma-Aldrich), and 98% *N*-Hydroxysuccinimide (NHS; 130672, Aldrich) were used to functionalize and activate the LSPR sensor surface. Rabbit anti-mouse IgG (ab46540, Abcam, Cambridge, UK) and natural mouse IgG protein (ab198772, Abcam) were selected as antibody and antigen pairs. 11-MUA was prepared in 95% ethanol and other reagents were prepared in 1X phosphate buffer saline (PBS) (10010-023, Gibco, Thermo Fisher Scientific, Waltham, MA, USA). The EDC, antibody and antigen pairs were stored at −20 °C, 11-MUA was stored at 4 °C and other chemical reagents were stored at room temperature.

### 2.4. Au Nanostructure Surface Functionalization and Immunoassay Protocol

To immobilize the antibody on the substrate, the nanostructure surface was first functionalized overnight with 0.01 M 11-MUA for approximately 12 h. Then, 11-MUA was activated by a 0.1 M EDC and 0.025 M NHS mixture at a 1:1 volume ratio in PBS for 30 min. After surface activation, 60 μL of 1000 μg mL^−1^ IgG antibody were loaded into the microchannel using the peristaltic pump at 10 μL min^−1^ for 15 min to ensure full immobilization on the surface. Finally, 60 μL of IgG at different concentration (5, 10, 20, 50, 100, 500, 1000 μg mL^−1^) were sequentially pumped into the microchannel under the same flow conditions for antibody-antigen interaction. Between each IgG concentration loading, the substrate was thoroughly washed by PBS at 10 μL min^−1^ for 10 min to remove any excessive solution or unbounded molecules. For one antibody-antigen binding event, the total assay time is 50 min (15-min antibody loading followed by 10-min PBS washing and 15-min antigen loading followed by another 10-min PBS washing).

## 3. Results and Discussion

### 3.1. Optical Images of Au Nanostructures under Various Fabrication Conditions

**Figure 1:**
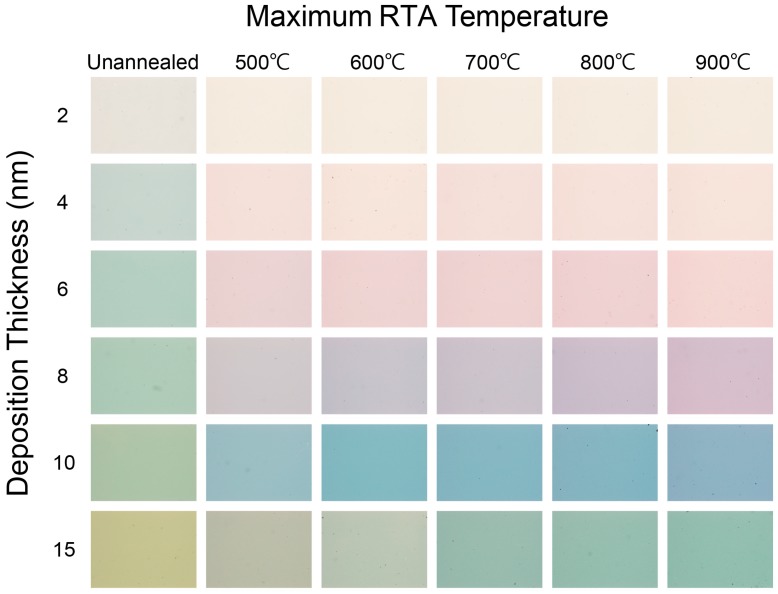
Images of physical vapor deposition (PVD) gold thin film with deposition thickness from 2 to 15 nm under 500 to 900 °C rapid thermal annealing (RTA) treatment. Each image is 2.2 mm × 1.6 mm.

Figure 1 shows images of the thin films with deposition thicknesses ranging from 2 nm to 15 nm and the maximum RTA temperatures ranging from 500 to 900 °C. The images of unannealed 2 to 15 nm gold films are also shown for comparison. All images were captured by a CCD camera (DFK 23U274, Imagesource, London, UK) attached to a stereoscopic zoom microscope. In each deposition thickness, the color of substrates subjected to RTA becomes darker with increasing maximum RTA temperature; the color of substrates gradually changes from light yellow to purple to green when the deposition thickness increased from 6 nm to 15 nm at 900 °C. The color change is due to the LSPR effect where the energy of incident light at the particular wavelength was absorbed by the substrates.

### 3.2. Optical Spectrum Measurement

**Figure 2:**
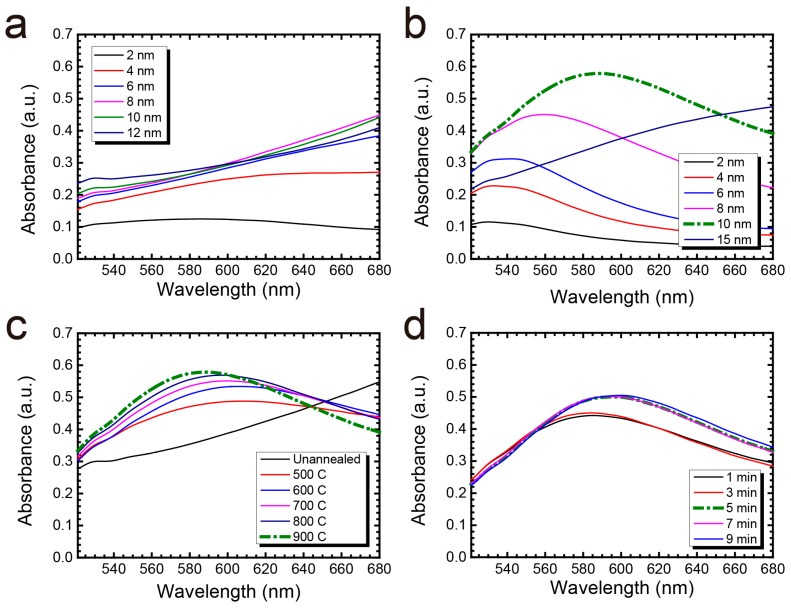
Change in absorbance spectrum under different annealing conditions: (**a**) Gold thin film thickness varying from 2 to 15 nm (**a**) without and (**b**) with RTA treatment; (**c**) 10 nm gold thin film with the maximum RTA temperature varying from 500 to 900 °C for 5 min; (**d**) 10 nm gold thin film under 900 °C RTA treatment with 1 to 9 min annealing time. Dashed line represents the optimal fabrication condition.

Ideally, the sharper the optical spectrum—represented by a narrower FWHM—the easier we can identify the LSPR peak wavelength shift due to molecules bound to the nanostructures. A narrower FWHM means a better figure of merit (FOM, the sensitivity factor divided by FWHM), which was preferred in our nanoplasmonic sensor. Another aim was to allow the LSPR peak wavelength of the sensor to fall in the visible wavelength band (500–700 nm in this work). If the LSPR effect occurs in visible wavelengths, we can observe its color change using the naked eye or a color camera. As shown in Figure 2a, the substrate without RTA could not induce the LSPR effect in the 500–700 nm wavelength range. After RTA, the LSPR peak can be clearly identified. As the deposition thickness increased from 2 to 15 nm, the FWHM and the absorbance spectrum became sharper and higher, respectively (Figure 2b). However, the LSPR peak wavelength for the 15 nm cases was out of the 500–700 nm range. Therefore, we selected the 10 nm deposition condition to evaluate the maximum RTA temperature and annealing time.

As to the annealing temperature condition, the absorbance spectrum became sharper, with a shift in peak wavelength toward the blue (607.59 to 588.07 nm) as the maximum RTA temperature increased from 500 to 900 °C (Figure 2c). For annealing time, the peak absorbance increased from 0.442 to 0.505 with a shift in the peak wavelength from 584.96 to 599.55 nm as the annealing time increased from 1 to 9 min (Figure 2d). However, the absorbance spectra were almost identical in the 5-, 7- and 9-min 900 °C RTA cases. Therefore, we fixed the annealing time at 5 min. Based on the above results, we conclude that a 10 nm gold deposition thickness with a 5-min 900 °C RTA treatment is the best fabrication condition for the subsequent immunoassay analysis.

### 3.3. Surface Morphology Analysis

**Figure 3:**
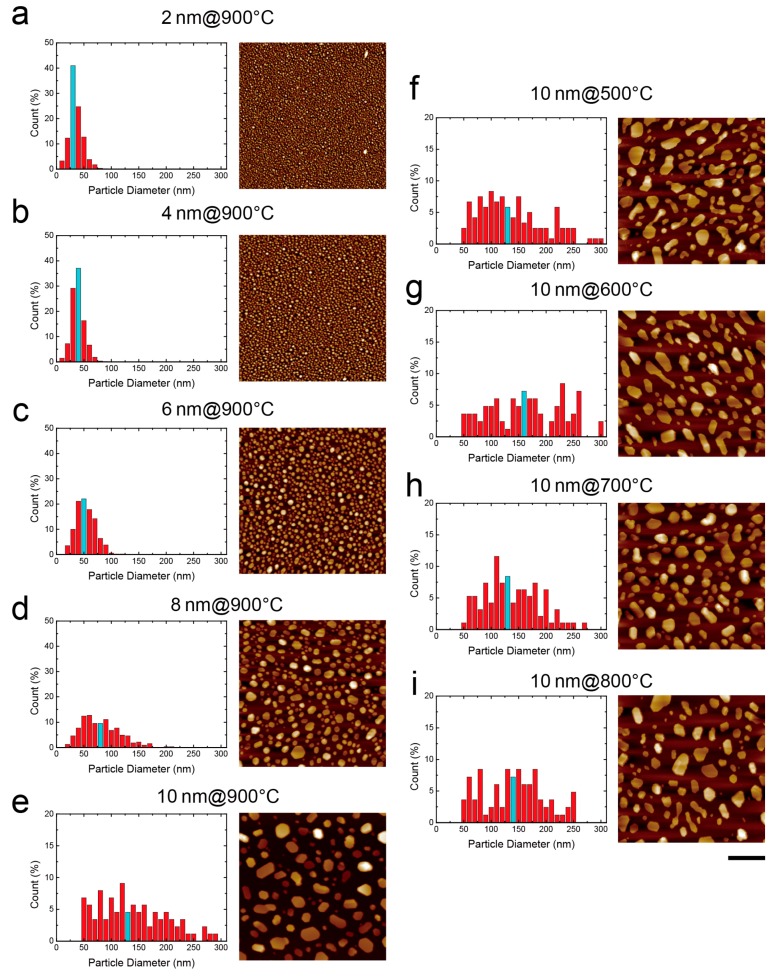
AFM images and histograms of RTA-treated Au nanostructures diameter distribution at (**a**–**e**) 2–10 nm deposition thickness with 900 °C RTA for 5 min and (**f**–**i**) 10 nm deposition thickness with 500–900 °C RTA for 5 min. Blue color bar represents the mean diameter of the gold nanoparticle. Scale bar is 500 nm.

To further investigate the relationship between the absorbance spectrum and the surface morphology of our Au nanostructures, we used AFM to scan the substrate under different fabrication conditions. To confirm the nanostructures was not directly generated during the PVD process, we scanned the 2 and 10 nm gold thin films before RTA. As shown in Figure S3, the surface roughnesses of both substrates were much lower than for the annealed cases (*R*_a_ = 1.44 and 1.56 nm at 2 and 10 nm, respectively), indicating uniform and smooth deposition quality. To quantify the surface profile, we used Image J software (ImageJ 1.51k, Bethesda, MD, USA) to analyze nanoparticles based on four parameters: (1) the total number of particles (*N*_p_); (2) the equivalent particle diameter (*D*_a_); (3) the surface roughness (*R*_a_) and (4) the total area of particles (*A*_p_). The detailed particle distribution histogram and structure characteristics can be found in Figure 3 and Table 1. As shown in Figure 3, the surface morphology of the gold thin films changed from a smooth surface to spherical or hummocky nanostructures after RTA. As the deposition thickness increased from 2 to 10 nm under 5-min 900 °C RTA, *N*_p_ per square micrometer decreased from 1103 to 31, *D*_a_ increased from 20.6 nm to 113.8 nm and *R*_a_ increased from 3.1 to 14.5 nm (Figure 3a–e).

On the other hand, as the maximum RTA temperature increased from 500 to 900 °C at 10 nm deposition thickness, *N*_p_, *D*_a_ and *R*_a_ did not change significantly (Figure 3e–i). Instead, as the annealing temperature increased, the shape of the nanoparticles became more circular due to stronger particle recrystallization. Such rounded particle shapes could lead to a shorter absorbance spectrum [25]. Also, *A*_p_ decreased from 34.5% to 33.1%, which may correlate to the increasing gaps between nanoparticles and cause the LSPR spectra to be shifted towards the blue. This phenomenon is known as the *plasmonic ruler* [26,27,28,29]. Based on the surface morphology results, we believe the rounded nanostructures and the increased particle distance both contribute to the blue shift of the LSPR spectrum observed in Figure 2c.

As shown in Table 1, we noticed that while the variation of equivalent particle diameter is large, the surface roughness and total area of particles are quite consistent. This demonstrates that RTA treatment would generate nanoparticles with various sizes but evenly distributed over the surface. Even though the variance in particle diameter leads to a flatten absorbance spectrum, the spectrums of the overall sensor is uniform, which can be validated by the result in Figure S4.

To confirm the reproducibility of RTA treatment process, we prepared five individual Au nanostructures with the same parameters (10 nm deposition thickness under 5-min 900 °C RTA) and scanned each surface profile using AFM microscopy. The result was shown in Table S1, indicating high reproducibility of our fabrication method.

### 3.4. Sensitivity and Uniformity Test

**Figure 4:**
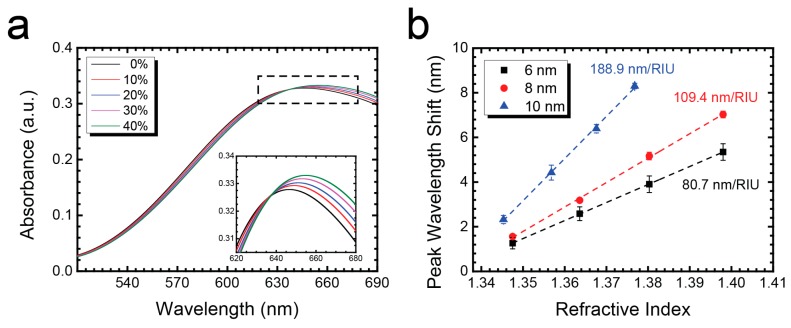
Sensitivity test of RTA-treated Au nanostructures. (**a**) Absorbance spectra of 0–40% glucose solution in DI water; (**b**) Sensitivity of 10 nm annealed Au nanostructures is 188.9 nm RIU^−1^ (*n* = 5).

Next, we characterized the sensitivity and uniformity of RTA-treated Au nanostructures by measuring their absorbance spectra. In the sensitivity test, 100 μL glucose solutions with serial dilution (10%, 20%, 30% and 40%) were pipetted into a 5-mm circular Polydimethylsiloxane (PDMS) well on top of an Au nanostructures under 5-min 900 °C RTA. The LSPR spectra of each glucose solution for 10 nm Au nanostructures are shown in Figure 4a. By plotting the LSPR peak wavelength shift of glucose solutions with corresponding refractive indices, we obtain the sensitivity factor (m, in nm per refractive index unit (RIU)) of the LSPR sensor. The refractive index of each glucose solution was measured by a refractometer (RA-130, KEM, Kyoto Electronics, Tokyo, Japan). As shown in Figure 4b, 10-nm Au nanostructures showed the highest sensitivity value (188.9 nm RIU^−1^). For comparison, we also conducted the sensitivity test for 6 nm and 8 nm Au nanostructures (109.4 and 80.7 nm RIU^−1^, respectively). The results confirmed that 10-nm nanostructures under 5-min 900 °C RTA have the highest sensitivity and are comparable to previous reported LSPR sensors made of Au nanostructures [30,31,32,33].

To test sensor uniformity, we measured the LSPR spectra in an air environment at different positions on the chip. Each detection spot was 600 μm in size and 1.25 mm away from another spot on a 10 mm × 10 mm LSPR substrate. The spectra are shown in Figure S4. Overall, the standard deviation of peak absorbance across locations was only 0.33 nm, which represents a very high uniformity of our LSPR sensor.

### 3.5. Multi-Point and Dynamic IgG Detection Using a Microchannel

**Figure 5:**
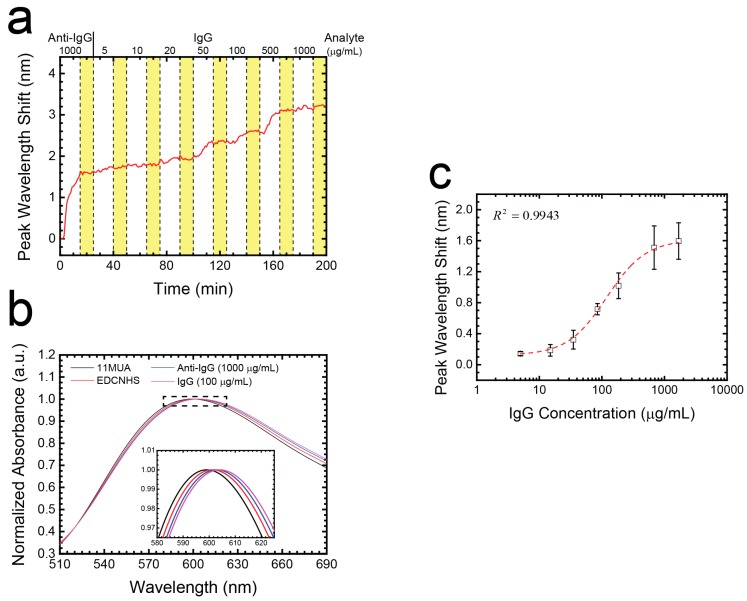
(**a**) Average of real-time multi-point IgG detection during LSPR sensor analyte detection process (*n* = 6). The yellow regions represent the 10 min of PBS washing separating each step; (**b**) Normalized LSPR spectra corresponding to IgG 100 μg/mL case processes in (**a**); (**c**) Purified IgG standard curve corresponding to the accumulation of the seven IgG concentration cases in (**a**).

To demonstrate the label-free and multi-point analyte detection capability of this nanoplasmonic sensor, we pumped purified IgG protein into a microchannel attached to the RTA-treated Au nanostructures. The channel size was 17 mm × 3.8 mm with a height of 0.4 mm. The total channel volume was 30 μL. For IgG detection, the substrate surface was first functionalized with 11-MUA and then activated with a mixture of EDC and NHS. Then the surface was immobilized with anti-IgG as the capture antibody. After surface functionalization, IgG at known concentrations (5, 10, 20, 50, 100, 500, and 1000 μg mL^−1^) was pumped into the channel in sequence from low to high concentration. Each concentration was continuously pumped for 15 min to enable full antibody-antigen interaction. Between each step, a PBS solution was loaded from the inlet into the device for 10 min to stabilize the background reagent condition, wash out unbounded molecules and prevent any nonspecific binding. Figure 5a shows the real-time multi-point IgG detection during LSPR sensor functionalization and the analyte detection process. Based on the above experimental protocols, we obtain the accumulated IgG concentration standard curve as shown in Figure 5c. The real-time wavelength shift curve was averaged by six sensing spots obtained in a single LSPR chip. With the concentration increasing from 5 to 1685 μg mL^−1^, the LSPR peak wavelength showed an increasing curve with a high r-squared when fitted to a logistic curve and a 1.595 nm total wavelength shift.

## 4. Conclusions

In summary, we have demonstrated a simple and cost-effective fabrication method using metallic PVD followed by RTA treatment to make a large-area nanoplasmonic sensor. To increase the sensitivity and uniformity of the sensor, we optimized the fabrication parameters, including the deposition thickness, the maximum RTA temperature and RTA time. Based on the AFM and UV-VIS spectrometer results, Au nanostructures with 10 nm deposition thickness under 5-min 900 °C RTA showed the highest LSPR sensitivity (188.9 nm RIU^−1^). By integrating the centimeter-size Au nanostructures with microchannels, we performed seven IgG concentration measurements in a single device, greatly reducing antibody consumption and minimizing potential antibody immobilization variance between different experiments. The spatial confinement of the microchannel reduced the required assay volume (60 μL) and assay time (less than 30 min for one antibody-antigen conjugation experiment with 10 detection spots). Moreover, we synchronized the spectrometer with a motorized stage, which allowed us to record the absorbance spectrum at 10 detection spots in one minute. This multi-point detection functionality can further be applied to multiplex analyte detection by immobilizing multiple antibodies in the substrate. Currently, the sensitivity of our sensor is slightly lower than others fabricated by EBL, NSL or NIL techniques. We believe the reason for this may be the flatten absorption spectrum caused by RTA-treat nanostructures with various nanoparticles sizes randomly distributed over the surface. We will further adjust fabrication conditions of RTA treatment, including the annealing temperature or deposition materials to improve the sensitivity. Overall, based on its real-time, dynamic and multi-point analyte detection capability, we believe this LSPR sensor integrated with a microchannel and a motorized stage has the potential to be applied to real-time and rapid clinically-based immunoassay studies.

## Figures and Tables

**Table 1:** Structural characteristics per square micrometer of RTA-treated Au nanostructures with different deposition thicknesses and the maximum RTA temperature.

**Maximum RTA Temperature (°C )**	**900**				
Deposition thickness (nm)	2	4	6	8	10
Number of particles (*N*_p_)	1103 ± 21	785 ± 22	281 ± 15	98 ± 14	31 ± 4
Equivalent particle diameter (*D*_a_) (nm)	20.6 ± 6.9	25.4 ± 7.9	39.9 ± 15.6	61.7 ± 29.4	113.8 ± 53.7
Surface roughness (*R*_a_) (nm)	3.1 ± 0.0	5.2 ± 0.2	8.7 ± 0.2	11.9 ± 0.5	14.5 ± 0.6
Total area of particle (*A*_p_) (%)	40.4 ± 0.4	43.4 ± 0.1	39.7 ± 0.5	33.6 ± 0.6	33.1 ± 1.3
**Deposition Thickness (nm)**	**10**				
Maximum RTA temperature (°C)	500	600	700	800	900
Number of particles (Np)	43 ± 5	32 ± 4	36 ± 3	31 ± 5	31 ± 4
Equivalent particle diameter (Da) (nm)	97.1 ± 48.1	114.0 ± 49.4	99.9 ± 47.4	107.1 ± 50.4	113.8 ± 53.7
Surface roughness (Ra) (nm)	12.9 ± 0.2	13.6 ± 0.2	14.4 ± 0.5	14.0 ± 0.8	14.5 ± 0.6
Total area of particles (Ap) (%)	34.5 ± 2.0	33.7 ± 1.2	31.1 ± 0.3	29.5 ± 1.3	33.1 ± 1.3

## Supplementary Materials

The following are available online at http://www.mdpi.com/2079-4991/7/5/100/s1. Figure S1: Schematic diagram of the LSPR sensing platform. Microchannel dimension is 17 × 3.8 mm with 0.4 mm channel height. LSPR spectra at 10 different positions can be sequentially on the sensor. Each detection spot has a diameter of 600 μm and is 1.25 mm apart from other spots, Figure S2: *x*^8^ polynomial curve-fitting algorithm to fit raw spectrum data. The spectral resolution of is 0.0558 nm and the noise level is only 1.18%, Figure S3: AFM images of (a) 2 nm and (b) 10 nm gold thin film without RTA treatment. Scale bar is 500 nm, Figure S4: Absorbance spectrum uniformity of LSPR sensor. The standard deviation is 0.33 nm (*n* = 5). Table S1: AFM images and structural characteristics per square micrometer five independent chips with deposition thickness of 10 nm under 5 min, 900 °C RTA treatment.
