# Supplementary material for: A Large-Area Nanoplasmonic Sensor Fabricated by Rapid Thermal Annealing Treatment for Label-Free and Multi-Point Immunoglobulin Sensing
Source: Nanomaterials (Basel). 2017 May 2;7(5):100. doi: 10.3390/nano7050100 (PMC5449981; doi:10.3390/nano7050100)
Supplement: Supplementary file 1 [file nanomaterials-07-00100-s001.pdf]

Supplementary Materials: A Large-Area Nanoplasmonic Sensor Fabricated by Rapid Thermal Annealing Treatment for Label-Free and Multi-Point Immunoglobulin Sensing

Hana Tzu-Han Lin, Chuan-Kai Yang, Chi-Chen Lin, Albert Meng-Hsin Wu, Lon A. Wang and Nien-Tsu Huang

Table S1. AFM images and structural characteristics per square micrometer five independent chips with deposition thickness of 10 nm under 5 min, 900 °C RTA treatment.

Chip #	1	2	3	4	5
AFM Images (size: 2 μm × 2 μm)	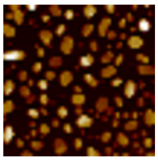	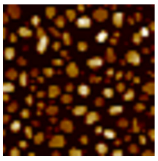	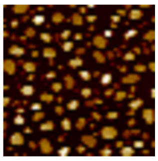	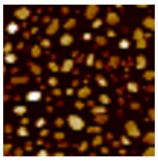	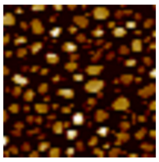
Number of particles (Np)	27 ± 5	28 ± 4	34 ± 4	31 ± 4	31 ± 2
Equivalent particle diameter (Da) (nm)	119.7 ± 57.5	118.1 ± 51.2	106.3 ± 51.4	109.8 ± 55.0	116.9 ± 48.9
Surface roughness (Ra) (nm)	14.6 ± 0.8	14.2 ± 0.5	14.0 ± 0.4	14.5 ± 0.5	14.1 ± 0.6
Total area of particle (Ap) (%)	33.2 ± 0.7	33.8 ± 1.8	33.0 ± 1.1	32.1 ± 1.5	34.0 ± 1.1

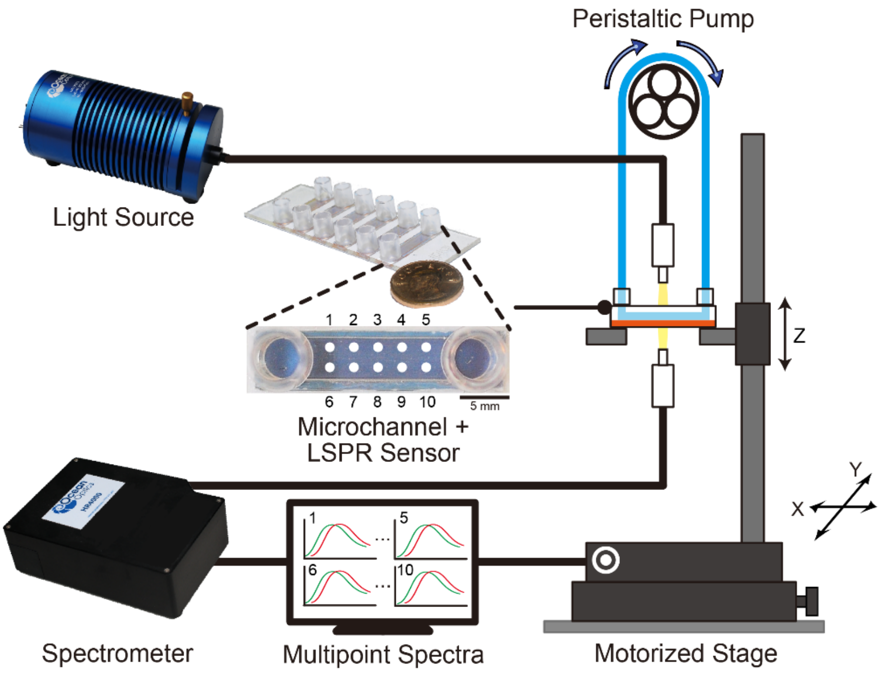

Figure S1. Schematic diagram of the LSPR sensing platform. Microchannel dimension is 17 × 3.8 mm^2 with 0.4 mm channel height. LSPR spectra at 10 different positions can be sequentially on the sensor. Each detection spot has a diameter of 600 μm and is 1.25 mm apart from other spots.

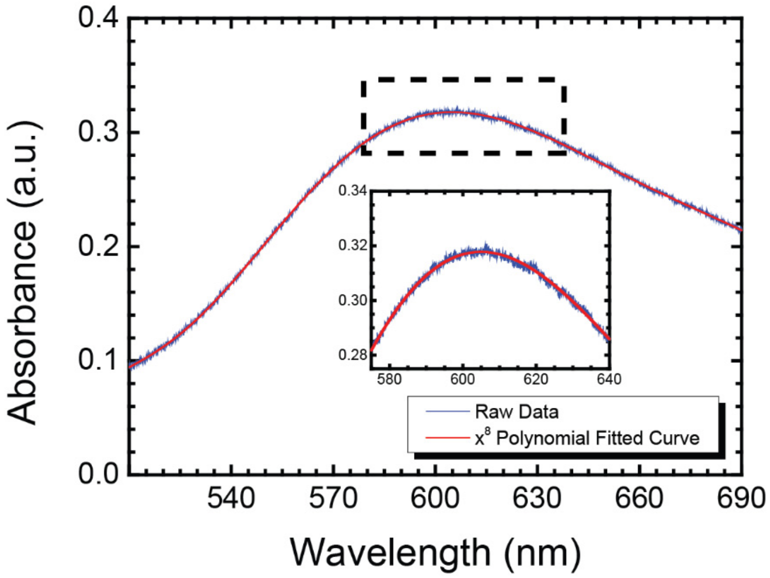

Figure S2. x^8 polynomial curve-fitting algorithm to fit raw spectrum data. The spectral resolution of is 0.0558 nm and the noise level is only 1.18%.

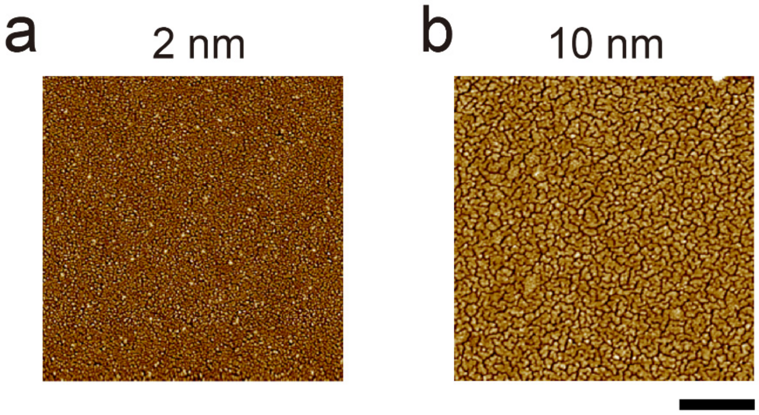

Figure S3. AFM images of (**a**) 2 nm and (**b**) 10 nm gold thin film without RTA treatment. Scale bar is 500 nm.

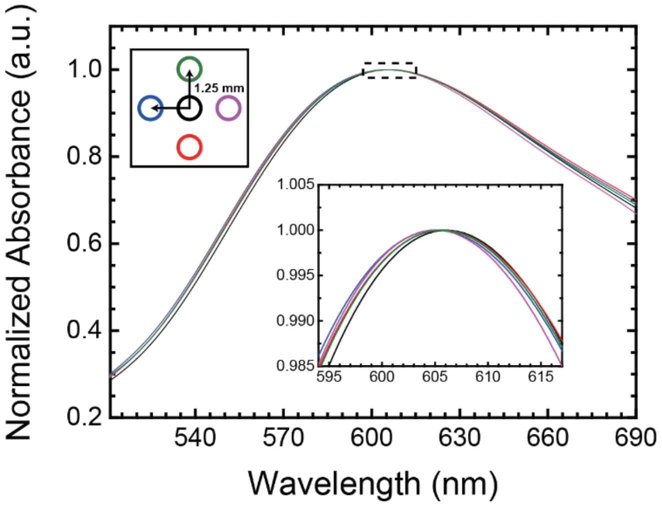

Figure S4. Absorbance spectrum uniformity of LSPR sensor. The standard deviation is 0.33 nm (n = 5).
